# Supplementary material for: Identification of photoperiod-induced specific miRNAs in the adrenal glands of Sunite sheep (Ovis aries)
Source: Front Vet Sci. 2022 Jul 22;9:888207. doi: 10.3389/fvets.2022.888207 (PMC9354845; doi:10.3389/fvets.2022.888207)
Supplement: Supplementary file 1 [file Data_Sheet_1.ZIP › Supplementary file/miRDeep2 file.zip!/miRDeep2 file/Know_Structure/SPLP42A3/oar-mir-323c.pdf]

| | |
|---|---|
| miRBase precursor | : oar-mir-323c |
| Total read count | : 96 |
| oar-miR-323c read count | : 95 |
| remaining reads | : 1 |

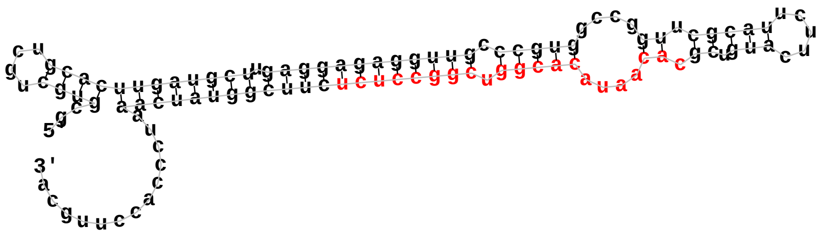

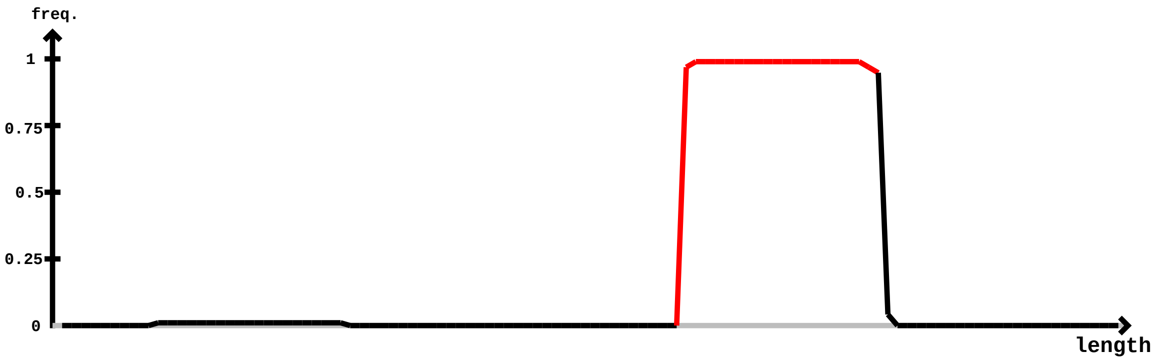


oar-miR-323c

```
5'- gcgugcugcugcacuugaugcuugaggagagguugcccguggccgguucgcauucuucaugucgcacaauacacggucggccucucuucgguaucaaaucccaccuugca   -3'    exp
    ..((((....))))((((((((.((((((((((((.(((((....((.(((((.....))).)).)).))....))))).))))))))))))))))))............    reads     mm        sample
    .........ugcacuugaugcuugaggag.................................................................................    1         0         seq
    ................................................................cacaauacacggucggccu...........................    2         0         seq
    ................................................................cacaauacacggucggccuc..........................    2         0         seq
    ................................................................cacaauacacggucggccucu.........................    85        0         seq
    ................................................................cacaauacacggucggccucuc........................    4         0         seq
    .................................................................acaauacacggucggccucu.........................    2         0         seq
```
